# Prevalence and associated factors of tuberculosis/HIV coinfection among people living with HIV in Almaty, Kazakhstan: a cross-sectional study

**DOI:** 10.3389/fpubh.2026.1816007

**Published:** 2026-08-19

**Authors:** Alfiya Y. Denebayeva, Elizabeth J. King, Zhamilya S. Nugmanova, Ulyana A. Gubareva, Marat S. Tukeyev, Anarkhan K. Nurkerimova, Aigerim B. Alimbekova, Elena Y. Ushanskaya, Roberta Z. Horth, Alexander J. Millman, Dilyara A. Nabirova

**Affiliations:** 1 Almaty City AIDS Сenter, Almaty, Kazakhstan; 2 Asfendiyarov Kazakh National Medical University, Almaty, Kazakhstan; 3 Central Asia Advanced Field Epidemiology Training Program, Almaty, Kazakhstan; 4 School of Public Health, University of Michigan, Ann Arbor, MI, United States; 5 Division of Global Health Protection in Central Asia, United States Centers for Disease Control and Prevention, Almaty, Kazakhstan

**Keywords:** antiretroviral therapy, HIV, Kazakhstan, TB determinants, tuberculosis

## Abstract

**Introduction:**

Tuberculosis (TB) is the leading cause of death among people living with HIV (PLHIV). Kazakhstan faces a growing number of new HIV diagnoses alongside a high incidence of multidrug-resistant TB. This study aimed to determine the prevalence and risk factors for TB among PLHIV in Almaty, Kazakhstan.

**Methods:**

We conducted a cross-sectional study of adult PLHIV receiving care at the Almaty AIDS City Center by 2022. Participants were randomly selected from the HIV registry. TB/HIV coinfection was defined as a documented TB diagnosis following HIV diagnosis in the registry. Bivariable and multivariable Poisson regression with robust standard errors was used to estimate unadjusted and adjusted prevalence ratios (PR and aPR) and 95% confidence intervals (CI) for factors associated with TB/HIV coinfection.

**Results:**

Of 194 PLHIV randomly selected, 188 participated, of whom 32 (17%) had TB/HIV coinfection. In bivariable analysis, coinfection was more common among those with lower educational attainment (24% vs. 6%), a history of incarceration (43% vs. 11%), ever smoking (32% vs. 4%), known TB contact (28% vs. 5%), detectable viral load (>50 copies/mL; 42% vs. 3% among those with undetectable viral load ≤50 copies/mL), ART duration less than three years (13% vs. 1%), and ART interruptions (16% vs. 3%). Coinfection prevalence was also higher among those with a CD4 cell count <200 cells/mm^3^ (44% vs. 12%), and those with no or unknown TPT receipt had a higher prevalence of coinfection compared to those who had received TPT (25% vs. 10%; PR = 2.5; 95% CI: 1.2–5.5). In multivariable analysis, coinfection was independently associated with younger age (18–39 vs. ≥40 years; aPR = 2.0; 95% CI: 1.2–3.4), ever smoking (aPR = 3.6; 95% CI: 1.3–10.0), known TB contact (aPR = 2.4; 95% CI: 1.0–5.7), incarceration history (aPR = 1.8; 95% CI: 1.1–3.2), and detectable viral load (aPR = 6.9; 95% CI: 2.5–19.5).

**Conclusion:**

We found a high prevalence of TB/HIV coinfection and suboptimal TPT coverage among PLHIV in Almaty. Expanding TPT access, ensuring routine TB screening, and prioritizing early and sustained ART, particularly among individuals with detectable viral loads or low CD4 counts, may help reduce TB among PLHIV in similar settings.

## Introduction

In 2022, an estimated 10.6 million people developed tuberculosis (TB) and 1.3 million died from the disease globally. TB caused 167,000 deaths among people living with HIV (PLHIV) worldwide. PLHIV are 14–20 times more likely to develop TB disease and die from TB than those without HIV (1). Understanding the factors associated with incident TB among PLHIV is essential for designing effective prevention and intervention strategies. Studies have shown that TB risk is influenced by a low CD4 cell count, high HIV viral load (2, 3), tobacco use, advanced WHO HIV clinical stage, and comorbidities such as diabetes mellitus (4–6) and anemia (7, 8). Conversely, early antiretroviral therapy (ART) initiation and good adherence have been found to significantly reduce TB risk (9).

Kazakhstan, with TB incidence of 49 per 100,000 population, is among the 30 high-burden countries for multidrug-resistant TB. In 2023, one-third (32%) of 3,855 people newly diagnosed with TB in the country had multidrug- or rifampicin-resistant TB (10). Consistent with trends in Eastern European and Central Asian countries, the HIV prevalence in Kazakhstan increased from 0.27% in 2020 to 0.33% in 2024 (11). However, only 82% of PLHIV in 2019 were aware of their status, and about 600 people were co-treated for TB and HIV (10).

In Almaty city the largest city in Kazakhstan, HIV prevalence among people aged 15–49 was 0.48% in 2023 (12). In 2017, Almaty began offering prompt initiation of ART for people diagnosed with HIV. However, despite these recommendations, PLHIV with TB were often less likely to initiate ART (13). Similarly, the number of PLHIV on ART increased from 2,400 in 2018 to 3,848 in 2022, yet the number of TB cases among PLHIV has not declined (12). TB remains the leading cause of death among PLHIV in the city. In 2021, 21% of 76 PLHIV diagnosed with TB died.

Our study aimed to identify risk factors associated with TB coinfection among adult PLHIV and to assess the relationship between TB coinfection and sociodemographic, behavioral, and clinical characteristics among PLHIV under care at the Almaty City AIDS Center.

## Methods

### Design and study settings

We conducted a cross-sectional study among adult PLHIV attending the Almaty AIDS Center, a public healthcare facility serving as the primary site for HIV clinical care and laboratory services in Almaty, Kazakhstan’s largest city with a population exceeding 2 million. A cross-sectional design was chosen to efficiently estimate the prevalence of TB/HIV coinfection and its associated factors using existing registry and clinical data, as uniform person-time and care entry data were unavailable, precluding a cohort approach.

### Study population

We calculated the minimum sample size to detect an association between ART status and TB/HIV coinfection, assuming a coinfection prevalence of 30% among ART-naive PLHIV and 15% among those on ART (prevalence ratio ≈ 2.0), based on previously published estimates (14). Using a two-sided significance level of 0.05, 80% power, and a 1:1 allocation ratio, the minimum required sample size was 176, increased by 10% for missing data to yield a target of 194 participants.

The study included PLHIV aged ≥18 years registered at the Almaty AIDS Center who provided informed consent to participate. Using a simple random sample, 194 individuals were selected from 3,848 PLHIV registered between January 1998 and December 2021 and receiving care by the start of 2022. Six declined to participate, resulting in a final sample of 188.

### Ethics approval

Ethical approval was obtained from the Local Ethics Committee of Asfendiyarov Kazakh National Medical University in Almaty, Kazakhstan (Approval No. 1268, dated 22.12.2021). All the data were anonymized using unique, non-identifiable codes. Written informed consent was obtained from all participants.

### Data collection

Data were collected through in-person interviews conducted by trained physicians experienced in HIV care. The interviews were recorded using the KoboToolbox© platform. The questionnaire included sociodemographic factors (e.g., age, sex, education, income) and behavioral factors (e.g., smoking, alcohol and drug use, incarceration history). Clinical and laboratory data, including baseline CD4 count, HIV viral load, ART use, TB preventive therapy (TPT), and TB history, were extracted from the AIDS Center patient registry.

Viral load was classified as detectable (≥50 copies/mL) or undetectable (<50 copies/mL). For participants with a TB diagnosis, viral load and CD4 count were extracted from the most recent measurement at or before TB diagnosis; for those without TB, the most recent available measurement was used. The primary outcome, TB/HIV coinfection, was defined as a documented diagnosis of TB following HIV diagnosis in the registry.

### Statistical analysis

Data were cleaned and analyzed using R software (version 4.3.1). Behavioral variables (alcohol, tobacco, and drug use) were categorized as “ever” (currently or previously engaged), and “never”. Descriptive statistics summarized participant characteristics.

Bivariable Poisson regression was conducted to estimate unadjusted prevalence ratios (PRs) and 95% confidence intervals (CIs) for factors associated with TB/HIV coinfection. To identify factors independently associated with the outcome, a multivariable Poisson regression model with robust standard errors, estimated using the sandwich estimator (sandwich package in R), was constructed. Variable selection followed two approaches: age and sex were included *a priori* as established demographic confounders regardless of bivariable significance; behavioral and clinical variables (smoking, TB contact, incarceration history, HIV viral load, and TPT status) were included based on bivariable associations (*p* < 0.05) and epidemiologic relevance identified in prior literature. Multicollinearity among candidate variables was assessed using variance inflation factors prior to inclusion. Adjusted prevalence ratios (aPRs) and 95% CIs were estimated from the final model.

## Results

Of 194 individuals randomly selected from the 3,848 PLHIV registered at the Almaty AIDS Center, resulting in a final sample of 188 participants. Among these, 32 (17%) had TB/HIV coinfection. The majority were male (62%) and the median age was 39 years (range: 20–66) (Table 1). Most (62%) had a secondary education or less. One in five had a history of incarceration (19%) or drug use (20%), and nearly half (47%) were current smokers. Fifty-four percent reported known contact with a TB patient and 53% had ever taken TB preventive therapy (TPT). Most (91%) were on ART prior to their TB diagnosis, of whom 57% had been on ART for less than three years; 39% reported ART interruptions. Thirty-six percent had a detectable viral load (>50 copies/mL), and 14% had a CD4 cell count below 200 cells/mm^3^.

**Table 1:** Factors associated with TB among people living with HIV in Almaty, Kazakhstan – bivariate analysis.

Characteristic	Total column % *N* = 188	TB/HIV coinfection row % *n* = 32	PR (95% CI)	*p*
Sex				
Female	71 (38%)	7 (10%)	—	
Male	117 (62%)	25 (21%)	2.1 (1.0–5.4)	0.069
Age (years)				
18–39	95 (51%)	20 (21%)	—	
≥40	93 (49%)	12 (13%)	0.6 (0.3–1.2)	0.179
Educational level				
Higher than secondary school	72 (38%)	4 (6%)	—	
Secondary school and below	116 (62%)	28 (24%)	4.3 (1.7–14.7)	**0.006**
Body mass index				
Normal	132 (70%)	23 (17%)	—	
Underweight	12 (7%)	3 (25%)	1.4 (0.3–4.1)	0.556
Overweight	44 (23%)	6 (14%)	0.8 (0.3–1.8)	0.593
Ever incarcerated				
No	142(76%)	16 (11%)	—	
Yes	35 (19%)	15 (43%)	3.8 (1.9–7.7)	**<0.001**
Preferred not to answer	11 (6%)	1 (9%)	0.8 (0.1–3.9)	0.835
Current or previous drug use				
No	146 (78%)	19 (13%)	—	
Yes	38 (20%)	12 (32%)	2.4 (1.2, 4.9)	**0.016**
Preferred not to answer	4 (2%)	1 (25%)	1.9 (0.1–9.3)	0.525
Current or previous alcohol misuse				
No	165 (88%)	25 (15%)	—	
Yes	23 (12%)	7 (30%)	2.0 (0.8–4.4)	0.103
Current smoker				
No	100 (53%)	4 (4%)	—	
Yes	88 (47%)	28 (32%)	7.9 (3.1–26.9)	**<0.001**
Contact with a confirmed TB case				
No	87 (46%)	4 (5%)	—	
Yes	101 (54%)	28 (28%)	6.0 (2.4–20.4)	**<0.001**
History of pneumonia				
No	96 (51%)	10 (10%)	—	
Yes	23 (12%)	7 (30%)	2.9 (1.1–7.6)	0.029
Unknown	69 (37%)	15 (22%)	2.1 (1.0–4.8)	0.072
Ever received TPT				
Yes	100 (53%)	10 (10%)	—	
No or unknown	88 (47%)	22 (25%)	2.5 (1.2–5.5)	**0.016**
On ART before TB diagnosis				
No	16 (8.8%)	16 (100%)	—	
Yes	166 (91%)	13 (8%)	0.1 (0.04–0.16)	**<0.001**
Years of ART before TB diagnosis (*n* = 166)				
≥ 3 years	71 (43%)	1 (1%)	—	
< 3 years	95 (57%)	12 (13%)	8.9 (1.8–16.3)	**0.035**
Ever had ART interruptions (*n* = 166)				
No	103 (61%)	3 (3%)	—	
Yes	63 (39%)	10 (16%)	2.3 (1.1–4.7)	**0.024**
Viral load^**^				
≤ 50 copies/mL	121 (64%)	4 (3%)	—	
> 50 copies/mL	67 (36%)	28 (42%)	12.6 (5.0–42.7)	**<0.001**
CD4 count^**^				
< 200 cells/mm3	27 (14%)	12 (44%)	—	
≥200 cells/mm3	161 (86%)	20 (12%)	0.3 (0.1–0.6)	**<0.001**

PR = Unadjusted prevalence ratio, CI=Confidence interval, P=Pearson’s Chi-squared test; Fisher’s exact test; Bolded if *p* < 0.05. TPT = TB preventive therapy.

^*^Most recent measure before or at the time of TB diagnosis, or the most recent measure for those without a TB diagnosis.

TB/HIV coinfection was more common among participants with lower educational attainment (24% vs. 6% among those with higher education), a history of incarceration (43% vs. 11%), current smoking (32% vs. 4%), known TB contact (28% vs. 5%), detectable viral load (42% vs. 3%), and CD4 cell count below 200 cells/mm^3^ (44% vs. 12%). Coinfection was also more frequent among those on ART for less than three years (13% vs. 1%) and those reporting ART interruptions (16% vs. 3%).

In bivariable analysis, secondary education was associated with a 4.3 times higher prevalence of TB/HIV coinfection compared to higher education (95% CI: 1.70–14.7) (Table 1). A history of incarceration was associated with a 3.8-fold higher prevalence (95% CI: 1.9–7.7) and contact with TB patients was associated with a 6-fold higher prevalence (95% CI: 2.4–20.4). Drug use and smoking were also significantly associated with a higher prevalence of coinfection (PR = 2.4; 95% CI: 1.2–4.9 and PR = 7.9; 95% CI: 3.1–26.9, respectively). No prior or unknown TPT was associated with a 2.5 times higher prevalence of TB compared to those who received TPT (PR = 2.5; 95% CI: 1.2–5.5). ART use was associated with lower coinfection prevalence (PR = 0.1; 95% CI: 0.04–0.16; *p* < 0.001), as was a CD4 cell count ≥200 cells/mm^3^ (PR = 0.3; 95% CI: 0.1–0.6; *p* < 0.001) (Table 1).”

In multivariable analysis (Table 2), TB/HIV coinfection was independently associated with younger age (18–39 vs. ≥40 years; aPR = 2.0; 95% CI: 1.2–3.4; *p* = 0.009), ever smoking (aPR = 3.6; 95% CI: 1.3–10.0; *p* = 0.014), known TB contact (aPR = 2.4; 95% CI: 1.0–5.7; *p* = 0.049), incarceration history (aPR = 1.8; 95% CI: 1.1–3.2; *p* = 0.034), and detectable viral load ≥50 copies/mL (aPR = 6.9; 95% CI: 2.5–19.5; *p* < 0.001). Sex was not significantly associated with coinfection (aPR = 1.3; 95% CI: 0.7–2.4; *p* = 0.492). TPT status was not independently associated with coinfection in the adjusted model, whether unknown (aPR = 1.0; 95% CI: 0.5–2.1; *p* = 0.952) or never received (aPR = 1.7; 95% CI: 1.0–3.0; *p* = 0.076) compared to prior TPT receipt.

**Table 2:** Multivariable analysis of variables associated with TB/HIV coinfection, Almaty, Kazakhstan (*N* = 188).

Characteristic	aPR	95% CI	*p*
Male (*vs female*)	1.3	0.7–2.4	0.492
18–39 years old (*vs* ≥*40 years old*)	2.0	1.2–3.4	**0.009**
Ever smoked (*vs never*)	3.6	1.3–10.0	**0.014**
Had a TB contact (*vs no TB contact reported*)	2.4	1.0–5.7	**0.049**
Ever incarcerated (*vs never*)	1.8	1.1–3.2	**0.034**
HIV viral load* ≥ 50 copies/mL (*vs <50* copies/mL)	6.9	2.5–19.5	**<0.001**
Unknown TPT (*vs prior TPT*)	1.0	0.5–2.1	0.952
No TPT (*vs prior TPT*)	1.7	1.0–3.0	0.076

aPR = Adjusted prevalence ratio, CI=Confidence interval; P=Wald z-test *p*-value from Poisson regression with robust standard errors; Bolded if *p* < 0.05, TPT = TB preventive therapy.

^*^Most recent viral load before or at the time of TB diagnosis, or the most recent result for those without a TB diagnosis.

## Discussion

Approximately one in six adult PLHIV receiving care at the Almaty AIDS Center developed TB after their HIV diagnosis. Several factors, including high HIV viral load, low CD4 cell count, history of incarceration, smoking, and short duration of ART, were associated with a higher prevalence of coinfection. These findings are relevant for healthcare providers and public health practitioners seeking to reduce TB among PLHIV in Kazakhstan.

Not receiving ART was strongly associated with TB diagnosis in our study. We found that a higher prevalence of TB was associated with those who had never initiated ART, had been on ART for fewer than three years, or experienced interruptions in treatment. This finding is consistent with prior studies showing that sustained ART use has been associated with substantially lower TB occurrence in longitudinal studies (15–19), a finding consistent with, though not confirmed by, our cross-sectional data. In our study, nearly one in ten PLHIV had not initiated ART, and all (100%) of these individuals were diagnosed with TB. Since 2017 in Kazakhstan, universal ART should be initiated no later than 14 days from the date of diagnosis, regardless of the clinical stage of the disease or CD4 cell count; prior to this, ART was initiated only after the onset of advanced HIV or low CD4. As a result, PLHIV diagnosed before this time may have experienced significant immunosuppression before starting treatment, increasing their susceptibility to TB.

We also observed a high proportion of ART interruptions (39%). Interruptions in care can result in rapid increases in viral load and immune decline, increasing susceptibility to opportunistic infections and TB occurrence (20). This finding is consistent with the higher prevalence of TB among individuals with detectable HIV viral loads and low CD4 cell counts in our study. Interruptions in care can also result in the emergence of drug resistance, and recent studies in Kazakhstan have reported high prevalence of drug-resistant mutations among PLHIV on ART (21).

In our study, PLHIV who received TB preventive treatment (TPT) had an associated lower prevalence of TB coinfection compared to those without documented TPT receipt. However, given the cross-sectional design and absence of temporal data on TPT initiation, this association should not be interpreted as evidence of a protective effect. This may reflect historical policy limitations in Kazakhstan, where TPT was not universally offered to all PLHIV until 2011. The universal provision of TPT to all PLHIV at the time of HIV diagnosis has been adopted only since the release of the 2011 WHO guidelines (22), which introduced clear recommendations for preventive therapy in this population. Under current national guidelines, TPT is typically delivered as a six-month course of isoniazid (300 mg).

Our findings regarding the association between isoniazid-based preventive therapy and lower TB prevalence are consistent with studies from countries in Africa and Central Asia (17, 23), which have similarly demonstrated reduced TB occurrence among individuals who complete TPT. However, given the cross-sectional design, causal relationships cannot be inferred. We did not assess the temporal relationship between TPT use and specific TB exposures, which may have influenced the observed association. Some individuals who were diagnosed with TB after receiving TPT may have been newly exposed following the completion of preventive therapy, for example, during periods of incarceration, potentially attenuating the observed association.

A substantial proportion of the PLHIV in our study, nearly one-fifth, had a history of incarceration. This finding is notable given the persistently high incarceration rates in countries in Eastern Europe and Central Asia (24). Although these rates have declined modestly over the past decade, Kazakhstan’s incarceration rate of 275 per 100,000 population exceeds the global average of 146 prisoners per 100,000 population. Prisons in Eastern Europe and Central Asia are well-documented environments for TB transmission, including drug-resistant forms of the disease, largely due to overcrowding, poor ventilation, and prolonged close contact (24). Moreover, punitive approaches that disproportionately target vulnerable groups, including people who use drugs, further concentrate individuals at risk for HIV and TB within prison settings (25). In our study, incarceration was associated with TB/HIV coinfection; however, causality cannot be inferred. Interventions that reduce incarceration and expand access to TB prevention, screening, and treatment within correctional facilities may contribute to reducing TB burden among PLHIV and in the broader population (24).

Our findings align with numerous epidemiological studies and meta-analyses demonstrating that smoking is an independently associated with TB infection (26). A recent meta-analysis reported a pooled relative risk of 1.73 (95% CI: 1.46–2.04) (27), whereas the association observed in our study was more than three times higher. The prevalence of smoking in our cohort (45%) was also substantially higher than that of the general adult population in Kazakhstan (20%) (28). Smoking impairs pulmonary function and compromises immune defenses, thereby increasing susceptibility to TB infection. It may also be associated with higher likelihood of TB recurrence by enabling the persistence of *Mycobacterium tuberculosis* even after treatment. Importantly, in Kazakhstan, where multidrug-resistant TB presents a major public health challenge, cigarette smoking has been linked to increased bacterial drug resistance (29). Interventions aimed at reducing tobacco use among PLHIV may therefore contribute to lowering TB occurrence and improving overall health outcomes.

The association between younger age and higher TB prevalence in our study warrants attention. Younger PLHIV may have been more recently diagnosed with HIV and therefore had less cumulative exposure to ART, a pattern consistent with the shorter ART duration we observed among those with TB coinfection. Additionally, younger individuals in our cohort had higher rates of smoking, incarceration, and drug use — all of which were independently associated with TB in our analysis — suggesting that the age effect may be partially mediated by behavioral and structural risk factors. This finding underscores the importance of intensified TB screening, early ART initiation, and targeted behavioral support for younger PLHIV, who may represent a particularly vulnerable subgroup.

This study has several limitations. First, its cross-sectional design precludes causal inference and prevents assessment of whether identified associations changed over time. Second, although participants were randomly sampled from the AIDS Center registry, findings may not be generalizable to PLHIV in other settings or those not captured in the registry. Third, self-reported data on stigmatized behaviors such as smoking and incarceration may have introduced recall and social desirability biases, potentially underestimating their true associations with TB/HIV coinfection. Fourth, we lacked data on TPT timing, regimen, duration, and completion, as well as whether TB exposure or incarceration preceded or followed TPT initiation. This introduces nondifferential misclassification bias likely biasing estimates toward the null and underestimating TPT’s protective effect. Fifth, viral load and CD4 cell count were not measured at a uniform time point: among those with TB coinfection these values reflected the clinical state at diagnosis, whereas among those without TB they reflected the most recently available measurement, limiting their interpretability as directional predictors. Future longitudinal studies with extended follow-up would help better establish temporality, assess the durability of TPT’s protective effect, and confirm the robustness of these findings.

Despite these limitations, this study has several notable strengths. The use of comprehensive clinical and laboratory data from the AIDS Center registry reduces reliance on self-report for key clinical variables such as viral load, CD4 cell count, and ART status, enhancing the accuracy and internal reliability of these measures. Furthermore, reliance on routine programmatic data ensures that findings reflect real-world clinical conditions, strengthening their relevance to HIV program planning in similar settings.

## Conclusion

Our study found that TB coinfection among adults living with HIV in Almaty was associated with multiple clinical, behavioral, and structural factors, including low CD4 cell count, high HIV viral load, lack of or interrupted ART use, smoking, substance use, and history of incarceration. The high prevalence of risk behaviors, ART interruptions, smoking and incarceration in this cohort further underscores their potential contribution to TB and highlights the importance of integrating ART adherence support, smoking cessation, and harm-reduction services into routine HIV care. Overall, our findings are consistent with prior evidence suggesting that timely ART initiation and sustained treatment continuity may reduce TB/HIV coinfection, though the cross-sectional design of this study precludes causal inference. These associations nonetheless highlight potential targets for programmatic intervention, to be confirmed by longitudinal research in Kazakhstan.

## Data Availability

The datasets generated and/or analyzed during this study are not publicly available due to participant privacy and ethical restrictions. They may be available from the corresponding author upon reasonable request and subject to the required permissions.
